# International Peer-teaching: the LernKlinik Leipzig “Erasmus-Week” for Incoming Erasmus Students

**DOI:** 10.3205/zma001202

**Published:** 2018-11-30

**Authors:** Daisy Rotzoll, Stefanie Wiemer, Anja Zimmermann, Philipp Alex, Jürgen Meixensberger

**Affiliations:** 1 University of Leipzig, Medical Faculty, LernKlinik Leipzig, Leipzig, Germany; 2 University of Leipzig, Prorectorate Education and International Affairs, Tutoring-Kolleg, Leipzig, Germany; 3 University of Leipzig, Medical Faculty, Leipzig, Germany; 4 University Hospital Leipzig, Department of Neurosurgery, Leipzig, Germany

**Keywords:** international medical students, peer teaching, peer-assisted learning, medical education, tutor training, skillslab, practical clinical skills

## Abstract

**Introduction: **This project report describes the development, pilot phase, evaluation and implementation of a preparatory course week for incoming Erasmus medical students at the LernKlinik Leipzig, the Skills and Simulation Centre of the Medical Faculty, University of Leipzig.

**Project description: **The aim of this project is to prepare Erasmus students for their year abroad using peer-assisted teaching as the method of choice. Major intended outcomes were support of language and clinical practical skill competency development, as well as enhancement of integration among international and German-speaking peer tutors. The methodological framework of Ross and Cameron [1] was used in planning the Erasmus-Week. For planning the 2012 pilot project, a survey among Erasmus students of the academic year 2011/12 was performed. All succeeding cohorts were asked to participate in pre- and post-surveys which were analyzed quantitatively and qualitatively.

**Results:** Between 2012 and 2017, n=173 European medical students spent their Erasmus year in Leipzig. Of these, n=148 (86%) participated in the Erasmus-Week. The country of origin of most Erasmus students was Rumania (20,3%). Among the most positively received aspects of the Erasmus-Week, the active use of German for medical purposes and the use of peer-teaching as the method of choice to learn and repeat basic medical examination skills were mentioned. Students emphasized their wish for being able to participate in further course offers.

**Conclusions: **Offering a preparatory course week for incoming Erasmus students focusing on language and clinical practical skills training using peer-teaching as the method of choice may facilitate the integration of Erasmus students into their foreign medical curriculum. Further studies are underway to elucidate if these experiences influence later professional careers and choice of employment.

## Zusammenfassung

**Einleitung: **Der vorgelegte Projektbericht beschreibt die Entwicklung, Pilotierung, Evaluation und Durchführung einer vorbereitenden Kurswoche für Erasmus-Studierende der Humanmedizin in der LernKlinik Leipzig, dem Skills- und Simulationszentrum der Medizinischen Fakultät der Universität Leipzig.

**Projektbeschreibung: **Ziel des vorgestellten Lehrprojektes ist es, im geschützten Rahmen mittels Peer-teaching Methode Erasmus-Studierende auf ihr Auslandsjahr vorzubereiten. Das Augenmerk liegt dabei sowohl auf der sprachlichen als auch auf der klinisch-praktischen Ausbildung, aber auch auf der Integrationsförderung durch engen Kontakt mit deutschsprachigen studentischen Peer-Tutoren. Als methodischer Rahmen für die Planung der Erasmus-Woche diente die Zusammenstellung von Ross und Cameron [1]. Zur Planung des Pilotprojektes 2012 wurde eine Befragung der Erasmus-Kohorte des Jahrgangs 2011/12 durchgeführt. Die nachfolgenden Jahrgänge wurden gebeten, an einer Prä-Post-Befragung teilzunehmen; die Freitextangaben der Prä- und Post-Befragungen wurden quantitativ und qualitativ ausgewertet.

**Ergebnisse: **Insgesamt n=173 Erasmus-Studierende der Humanmedizin kamen im Zeitraum 2012-2017 nach Leipzig; von diesen nahmen n=148 (86%) an der Erasmus-Woche teil. Die meisten Erasmus-Studierenden kamen aus Rumänien (20,3%). Mit Abstand die häufigsten Nennungen zu den positiven Aspekten der Erasmus-Woche betrafen die aktive Auseinandersetzung mit der deutschen Fachsprache und die Wertschätzung des Peer-teaching Verfahrens, um grundlegende Untersuchungstechniken erlernen bzw. wiederholen zu können. Ein Ausbau und eine Intensivierung des Kursangebotes wurden gewünscht.

**Schlussfolgerungen:** Die Durchführung einer vorbereitenden Kurswoche für Erasmus-Studierende mit Fokus auf Erwerb von sprachlichen und klinisch-praktischen Fertigkeiten im Peer-teaching Verfahren kann den Einstieg und die Integration Erasmus-Studierender in den Studienablauf erleichtern. Inwieweit diese Erfahrungen auch Einfluss auf die spätere berufliche Ausrichtung oder den Tätigkeitsort der Erasmus-Studierenden haben, soll in weiteren Befragungen untersucht werden.

## Introduction

The Erasmus programme of the European Union (EU) is one of the most important exchange programmes for European students and is often quoted as a unique success story [1]. This oldest educational programme of the EU (the acronym standing for **E**u**R**opean Community **A**ction **S**cheme for the **M**obility of **U**niversity **S**tudents) was founded in 1987 and celebrated its 30^th^ anniversary in 1987. Between 1987 and 2017, Erasmus supported approximately 4.400.000 students. As early as in 1990, the “Länder” of the eastern part of Germany were integrated into the Erasmus programme, making it possible for the University of Leipzig to become part of the exchange scheme. In 1995-96, further institutional contracts between universities followed, ameliorating further exchange between European institutions of higher education. In 1987, Erasmus mobility was implemented between 11 EU member countries, namely Belgium, Denmark, Germany, Greece, France, Ireland, Italy, the Netherlands, Portugal, Spain and the United Kingdom. Further (western) European countries followed and in 1998-99, eastern European countries participated, namely Poland, Rumania, Slovakia, the Czech Republic, Hungary, Bulgaria, Estonia, Latvia, Lithuania and Slovenia.

The Erasmus programme was a major motor for internationalization and globalization in the European educational sector. A substantial influence could be seen on the European healthcare systems, leading to rising numbers of international healthcare personnel working in foreign countries, i.e. in Germany. Although the trend of rising mobility in the healthcare systems is continuing, only few German medical faculties offer structured programmes to support initial study integration of incoming Erasmus students in Germany. This lack of structured integration programmes can lead to study periods abroad where touristic aims of the stay outweigh the intended aims of the Erasmus programme [2]. For obtaining new input for the medical career in the home country, getting to know a foreign and unknown healthcare system and collecting new personal experiences there are essential.

Main aims of the Erasmus programme are the forming of a European identity, support of international cooperation, support of cultural openness and multilingualism as well as social integration and inclusion of foreign students in their European host country and university [3]. An essential factor to make this happen is having early and intensive possibilities to interact with German medical students at the respective universities. Acquiring interested and highly motivated German students willing to support incoming Erasmus students is essential. Publications on German medical faculty support programmes for international students (i.e. foreign medical students who come to Germany without a German university entrance qualification and who do not intend to finish their studies in Germany) shows that some support programmes exist, such as peer-led tutorials during preclinical studies or language courses (“German for medical students”) [4]. This cohort is by no means identical with incoming Erasmus students, though. Erasmus students arrive in Germany with prior knowledge and experiences in their field of study and plan to return to their home country usually after one year, aiming to finish their studies in their home country. In medicine, many foreign faculties propose their Erasmus applicants to go abroad in the clinical part of their education. This also leads to the fact that incoming Erasmus students have other priorities and wishes than international students planning to finish their complete medical education in Germany.

Since Erasmus students frequently return to their home country after 12 months and German faculties may depend on good preparation of these students by their home faculties prior to their stay abroad, German faculties tend to underestimate the necessity of preparing incoming Erasmus students for their stay abroad. This leads to the fact that Erasmus incomings are frequently part of regular lessons and courses at the receiving faculties with little focus on specific needs or wishes. Even if organizational supportive measures are functioning, a specific preparation of Erasmus incomings may lead to a more effective stay abroad, as well as higher student satisfaction after their return. The project introduced here was implemented with the aim of offering a structured preparation for Erasmus incomings at the Medical Faculty in Leipzig.

## Project description

Each year, around 20 to 40 Erasmus incoming students are received at the University of Leipzig, Medical Faculty. Official welcoming and possibilities to build contacts with medical students at the receiving faculty were limited. In this paper, the initiative for Erasmus incomings, initiated by peer student tutors of the Medical Skills and Simulation centre (LernKlinik Leipzig), their medical director (DR) and personnel responsible for student-tutor qualification in didactics (SW), is described, focusing on establishment, implementation, further development and evaluation of the project. The pilot project of October 2012 and the development of the course concepts for the years 2013 to 2017 are described.

### Project aim

Aim of this project was the preparation of international Erasmus incoming students for their year abroad, using peer-teaching as the teaching method of choice in a safe small-group environment. Special focus was set on language and practice of clinical practical skills in small groups to enable early interaction with German-speaking peer student tutors of the LernKlinik. Planning and implementation of the project was based on the suggestions of Ross and Cameron on peer-teaching methodology [1].

#### Student-tutor qualification

A grant of the Joachim-Herz-Foundation offering fellowships for innovation in higher education enabled the implementation of the project “Train the trainer – new pathways in medical education. A system for sustained didactic-methodological qualification of student tutors at the LernKlinik Leipzig” (granted to SW) in 2011 [https://www.stifterverband.org/lehrfellowships/fellowships-hochschullehre-fellows-2011 (accessed Nov. 22, 2017)]. A cooperation between the LernKlinik Leipzig and the Leipzig University-led student-tutor qualification centre enabled the development of a structured student-tutor qualification programme for the LernKlinik. This programme focused on development of competencies and organizational qualifications of the student-tutor team, leading to knowledge and experience transfer from one student-tutor generation to the next. Professional and didactic quality development is meanwhile maintained by this concept for all student-tutor courses at the LernKlinik where student peer-teaching is involved [5].

#### Student-tutor project team „Erasmus-Week“ 

Based on the experiences of the LernKlinik student-tutor qualification programme mentioned above, a project team (n=6) started its work to develop a peer-teaching concept for supporting incoming Erasmus students. The project team consisted of four LernKlinik peer student tutors. All members had completed the LernKlinik peer-student-tutor qualification programme, were part of the peer-student-tutor team for at least one year and had collected experiences abroad themselves, either as an exchange student from their high schools or as an outgoing Erasmus medical student. The medical director of the LernKlinik (DR) and personnel responsible for peer-student-tutor didactic qualification (SW) supported strategic planning and project alignment. Furthermore, two LernKlinik team members were involved in organizational and administrative planning.

#### Concept and preparation

As a concept basis for deciding on Erasmus-Week course compilation, a questionnaire among incoming Erasmus students of the academic year 2011-12 distributed at the end of their stay (August 31, 2012) was used (n=20, returns 100%). The questionnaire was distributed electronically and evaluated with EvaSys^®^. 24 items were captured, giving data on gender, country of origin, length of medical education and stay in Germany, i.e. the city of Leipzig, as well as German everyday language and German medical language ability. Furthermore, information was obtained on medical course compilation during their Erasmus year as well as on their wishes and ideas about possible preparatory courses based on their experiences in Leipzig.

Questionnaire results and orally conveyed comments of medical faculty involved in clinical teaching of Erasmus incomings were used to decide on the compilation of peer-student-tutor led LernKlinik courses to be adapted for use in the initial Erasmus-Week in 2012.

The framework “24 Questions about Peer-Assisted Learning” by Ross and Cameron [1] served as the basis for developing the Erasmus-Week using peer-teaching as the method of choice.

#### Learning objectives and methodological implementation 

Ten 90-minute sessions held on 5 consecutive days, given for small groups of n=4-6 participants, under supervision of a professionally and didactically trained peerstudent tutor, were compiled. Each course was documented on a workflow sheet for tutor preparation, where time frame as well as learning objectives and teaching methods were documented. Defined learning objectives were documented (see Table 1 (Tab. 1) and Table 2 (Tab. 2)). For each course, a preparatory student manual for participants and a manual for peer-student-tutor use were compiled. The tutoring manuals aimed at giving hints and comments for specific situations possibly arising in the Erasmus-Week courses. Erasmus courses to be compiled were based on the courses for German medical students published before [6]. All learning materials were revised for implementation in the Erasmus-Week. To be prepared for possibly occurring language problems leading to increased course length, the learning objectives per course were restricted to a maximum of three. As supplements, vocabulary and frequently-used phrase lists (English-German) were added to the Erasmus course material, which was handed out to students in advance and displayed as posters in the course rooms for reference. 

#### Planning

The ideal time point for scheduling the Erasmus-Week was identified at the beginning of the winter term (mid-October of every year). All incoming Erasmus students have arrived by then, and an overlap with language courses offered by the university in September could be excluded. A team of approximately 16 peer student tutors met in July; all team members were available during mid-October. In accordance to participation lists of Erasmus incoming students for obligatory bedside-teaching courses in 2010 and 2011, 10 LernKlinik peer-student-tutor courses were selected, matching the disciplines that most Erasmus incoming students selected. Course selection was adapted from 2012 onward according to the needs assessment in the evaluations received. The following seven courses were therefore routinely offered every year: medical history-taking, heart auscultation, lung auscultation, examination of the abdomen, neurological status, paediatric precautionary examinations U1/U2, and the gynaecological precautionary examination. Three further courses were selected and changed respectively every year, depending on the cumulative evaluation results of the prior cohorts of Erasmus incomings and peer-student-tutor availabilities. These were: overall status examination, status of the thorax, examination of head and neck, orthopaedic examination of spine and hip, knee joint examination, obstetric examination, venipuncture and basic life support. Since 2017, the course for examination of the abdomen is held together with simulated patients, enhancing the linguistic challenge for the participants. The simulated patients involved are trained to give a specific feedback regarding spoken language comprehension.

The Erasmus-Week began with a welcoming by the dean of education (JM) and the medical director of the institution (DR) and finished with a get-together at the end of the week, making room for an informal exchange between LernKlinik peer student tutors and Erasmus incoming students. 

#### Group allocation

All Erasmus incoming students were divided into small groups with 4 to 6 participants each; the group allocation remained the same throughout the Erasmus-Week for all 10 courses. For group allocation, special care was taken to mix nationalities, so that German was the spoken language of choice. For alleviation in case of communication difficulties, German-English vocabulary and phrase lists were displayed as posters on the wall.

#### Evaluation

Prior to and after each Erasmus-Week, pre- and post-evaluation questionnaires were offered using EvaSys®. Prior to the course week, an 18-item-questionnaire in English was handed out, asking for information concerning personal data, pre-qualifications, choice of stay and course participation. Two items had free text answer sections. After the course week, a post-evaluation was distributed, containing 25 items on the topics language ability, course structure and quality, course materials and overall comments (3 free text sections). All free text sections of the pre- and post-questionnaires were evaluated qualitatively. In accordance with the evaluations, adaptations for the Erasmus-Week of the following year were made.

## Results

The following section gives an overview of the most important evaluation results regarding the Erasmus-Week in Leipzig.

### Preliminary investigation at the end of the year abroad: Erasmus incoming students of the academic year 2011/12

Gender distribution of Erasmus incoming students of the academic year 2011/12 (n=20) was approximately the same as the gender distribution among local students of medicine: 65% were female. About half the cohort originated from eastern European countries, while the other half came from western EU nations. 47% of the participants were in their 4^th^ year of medical studies, showing a wide range between 2^nd^ and 5^th^ year of education. 85% were completing their first study period abroad at a German medical faculty. 80% stayed at the medical faculty of Leipzig for 2 semesters (one year), 20% for one semester only. The length of prior German language instruction varied considerably, ranging from one month to over 9 years. About half the cohort participated in a German intensive language course for foreign students at the University of Leipzig prior to their Erasmus stay at the Medical Faculty. Main reasons for planning an Erasmus stay in Leipzig were: getting to know a foreign country, coming into contact with the German healthcare system, learning clinical practical skills and learning the German language. The three main reasons why Leipzig was the city of choice were: recommendations by prior Erasmus incoming students coming to Leipzig, Leipzig’s image as a student-friendly city and cheap living costs. The Erasmus incoming students hoped for a more intensive contact with German medical students and for a specific preparation for bedside-teaching courses.

Erasmus incoming students in their 2nd to 5th year of medical studies compile their own study curriculum in accordance with the education deanery and can choose their schedule from all courses offered. After returning to their home institution, credits can be recognized via the ECTS-system. With this method, Erasmus incoming students can compile their individual curriculum out of the course plans of the 3^rd^ to 5^th^ year of medical education in Leipzig and can align their plan with their individual needs. When asked from which disciplines practical bedside teaching courses were selected, 80% specified internal medicine, 70% paediatrics, 70% surgical disciplines and 60% obstetrics and gynaecology.

#### Erasmus incoming students participating in the Erasmus-Weeks of 2012-2017 

N=173 Erasmus incoming medical students came to Leipzig between the years 2012 and 2017; of these, n=148 (86%) participated in the Erasmus-Week.

**Figure 1:**
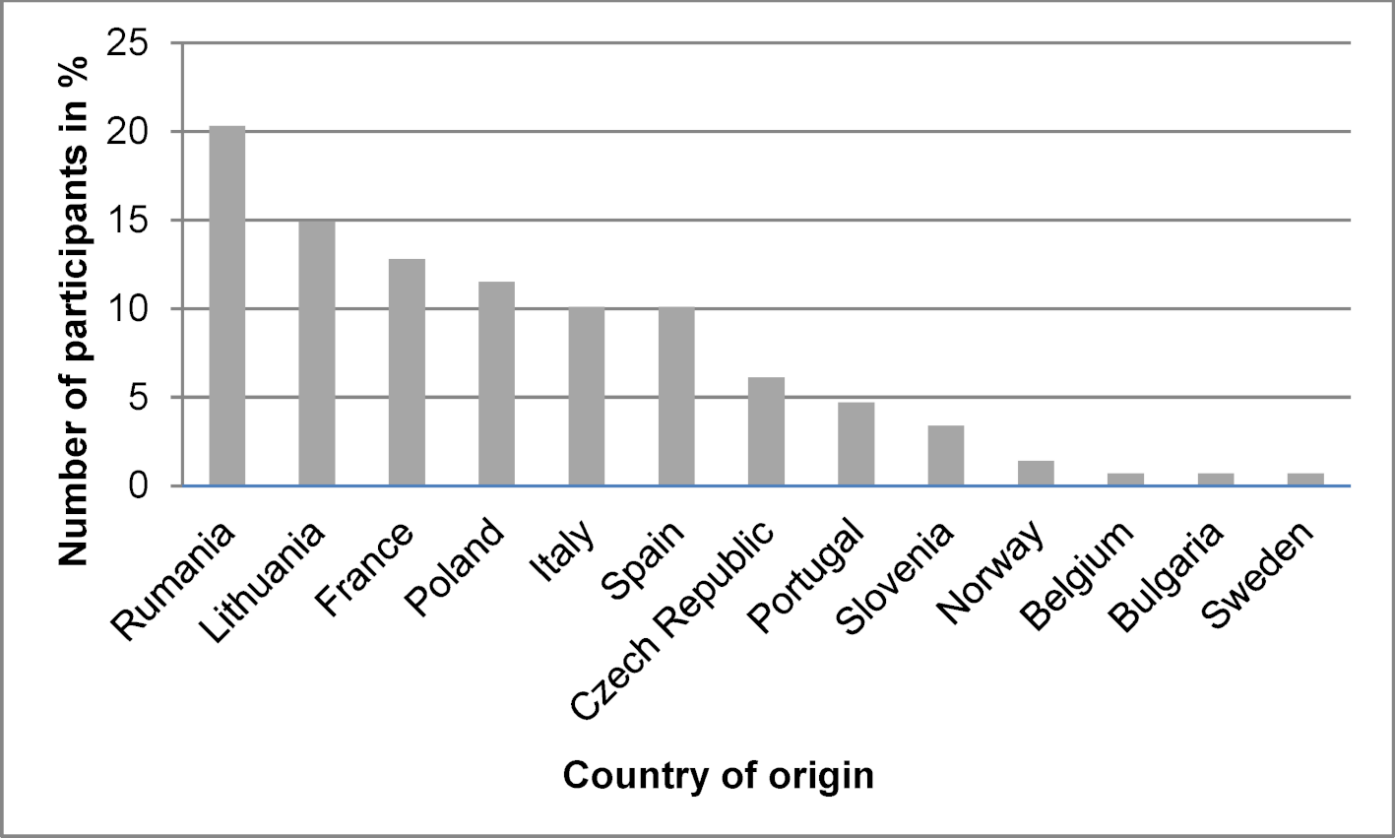
Leipzig Erasmus-Week participants, distribution by nation of origin, 2012-2017 (6 cohorts, n=148 in total)

The distribution by nationality of Erasmus incoming students is shown in Figure 1 (Fig. 1): the largest group originated from Rumania, followed by Lithuania, France and Poland. Out of the ten most frequently represented nationalities five were eastern European (Rumania, Lithuania, Poland, Czech Republic, Slovenia) and five were western European (France, Italy, Spain, Portugal, Norway).

Gender distribution of Erasmus-Week participants (n=148) showed a clear shift towards female participants (excluding the year 2014). 71,4%-77,4% of all participants were women.

#### Standardized evaluation tool – EvaSys^®^, qualitative analysis of free text answers 

Filling out the standardized pre- and post-questionnaires before and after Erasmus-Week participation was voluntary and anonymous. Among the n=148 participants, n=143 (97%) filled out the pre-questionnaires, while n=126 (85%) participated in the post-survey. Aims of the pre-evaluation were to obtain expectations and wishes concerning the LernKlinik Erasmus-Week, while the post-questionnaire aimed at data on satisfaction with the course offers (“If you had a wish open on what would make our courses even more useful for you, what would this be?”; “Do you think this course week should be repeated for future incoming Erasmus students in Leipzig, and if so, why?”; “Free comments: anything else you would like to tell us?”).

Table 3 (Tab. 3) summarizes the open-question results of the pre- and post-questionnaires. Three main repetitive themes were filtered out of the pre-evaluation (n=41):

1. gratefulness and high anticipation/curiosity concerning the stay abroad in general and specifically concerning the Erasmus-Week (n=29),
2. worries that language capability may not be sufficient for studies at the medical faculty of Leipzig (n=7), and
3. uncertainty if medical knowledge and individual preparation for the Erasmus stay abroad are sufficient (n=5).

Analysis of post-questionnaires showed recurrent themes as well. All themes that recurred at least twice are summarized in Table 3 (Tab. 3).

By far the most frequently mentioned nominations on positive aspects of the Erasmus-Week focused on active use of German medical language and appreciation of peer-teaching as a didactic method to learn or repeat basic examination skills. The two most frequently mentioned proposals for improvement concerned student wishes for broadening and intensifying the course week.

**Figure 2:**
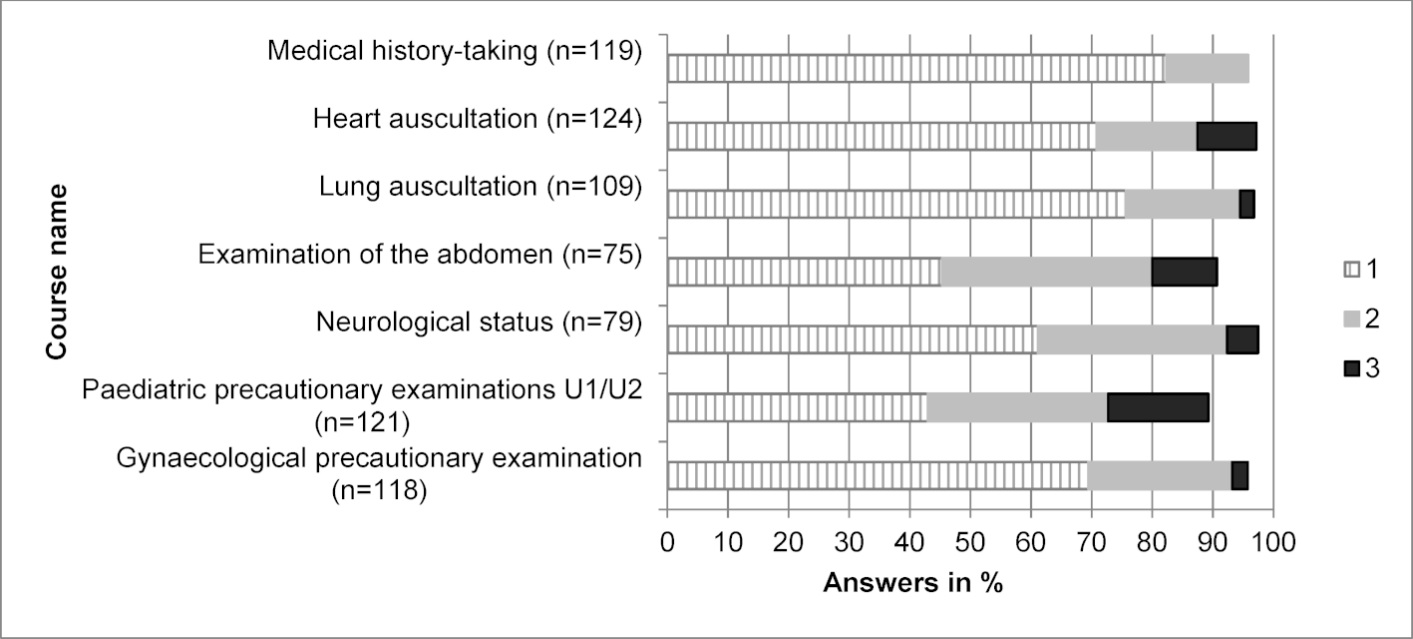
School grade distribution (1 – very good, 2 – good, 3 – satisfactory) of the 7 Erasmus-Week courses offered every year in 2012-2017 (6 cohorts, n=148). N in brackets corresponds to the number of evaluations for each course, respectively.

Figure 2 (Fig. 2) shows evaluation results of the seven Erasmus-Week peer-teaching courses given each year from 2012-2017, namely medical history-taking, heart auscultation, lung auscultation, examination of the abdomen, neurological status, paediatric precautionary examinations U1/U2 and gynaecological precautionary examination (with school grades, respectively). In over 70% of the answers, the courses in medical history-taking, heart auscultation and lung auscultation were marked with the school grade 1 (very good).

**Figure 3:**
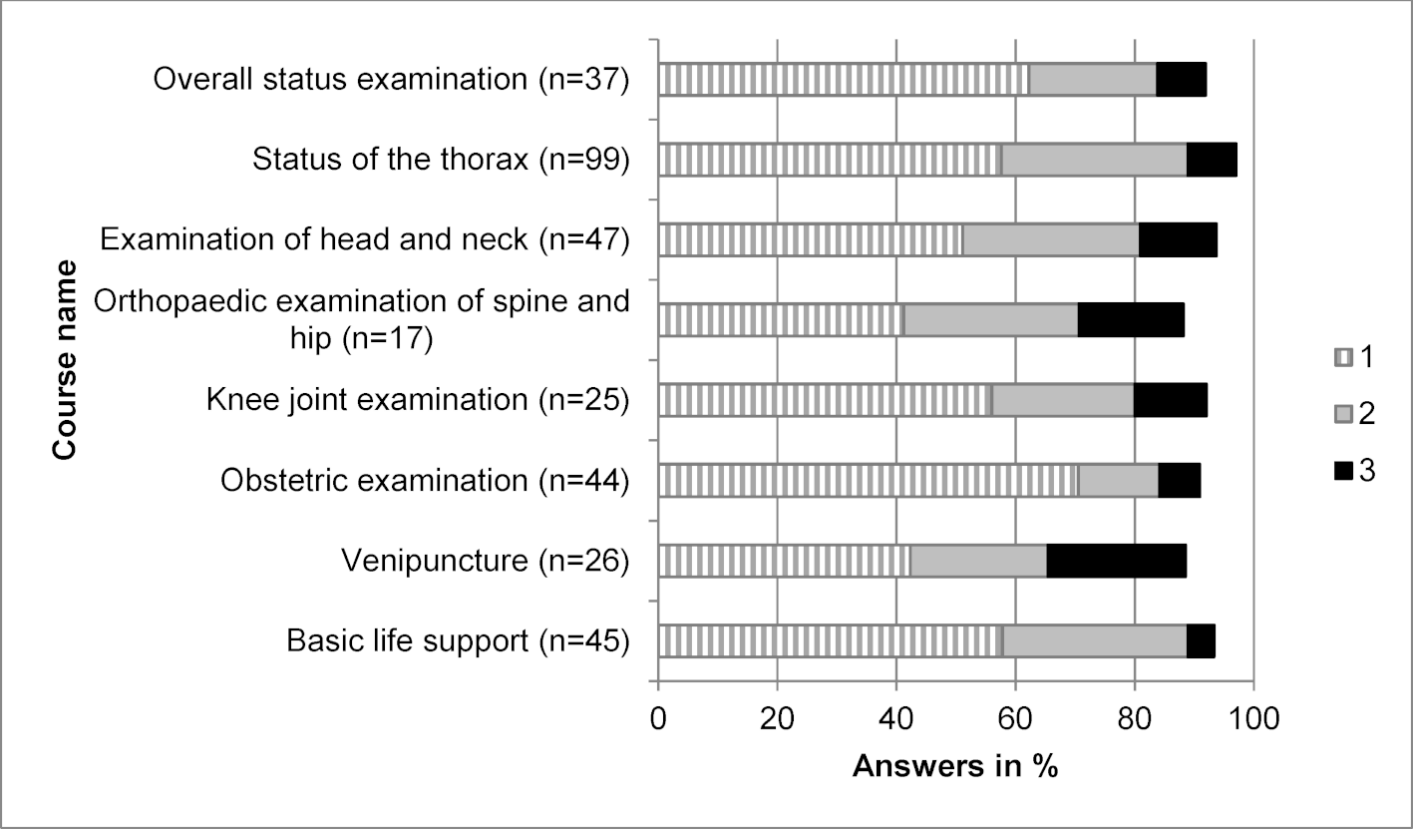
School grade distribution (1 – very good, 2 – good, 3 – satisfactory) of the Erasmus-Week courses offered intermittently in 2012-2017 (6 cohorts, n=148). N in brackets corresponds to the number of evaluations for each course, respectively.

Three yearly changing courses were offered additionally to the seven regular courses (see Figure 2 (Fig. 2)) to adapt the course portfolio according to the wishes of the prior cohorts. Figure 3 (Fig. 3) shows the evaluation results of the intermittently offered courses, namely overall status examination, status of the thorax, examination of head and neck, orthopaedic examination of spine and hip, knee joint examination, obstetric examination, venipuncture and basic life support, in school grades. A “very good” (school grade 1) in over 70% was only achieved by the course in obstetric examination.

## Discussion

Evaluation of the Erasmus-Week questionnaires of the years 2012-2017 gave important hints as to how the course week should be developed in future; these are discussed in the following.

### Participants of the Erasmus-Week

So far, n=148 medical students from 13 European countries participated in the course week described; during the survey period, a slight shift from a predominance of eastern European students to a more or less balanced number between eastern and western Europeans could be seen. The initially documented preference of eastern European medical students to choose Leipzig as the city of choice for their Erasmus year in Germany is no longer evident. Verbal communication from the medical education dean’s office shows that there seems to be a trend for western European students (i.e. from France) to commence an Erasmus year in Leipzig early on in their educational career (3rd year of medical school), while eastern European students (i.e. from Rumania) prefer to come at the end of their education. LernKlinik peer student tutors report that language competencies among Erasmus incoming students are extremely divergent, although all students are certified to be at a B2-level on the basis of the Common European Framework of Reference for Languages [http://www.europaeischer-referenzrahmen.de/ (accessed Nov. 22, 2017)]. Eastern European students seem to bring along better German language competencies. This fact led to the question of how group allocation during the Erasmus-Week should be performed. The peer-student-tutor project team had explicitly decided to mix nationalities in the groups to enforce German as the language of communication. On the other hand, results of the post-questionnaires (see Table 3 (Tab. 3)) showed that some Erasmus incoming students would have preferred to be allocated according to their ability to communicate in German. To take this wish into account, testing of language ability would have been necessary. Due to the fact that results of the pre-questionnaires showed student worries about possible insufficient linguistic and medical knowledge for their stay abroad, and that an entrance examination may have reinforced these fears, testing as an option was abandoned and mixing of nationalities for group allocation was preferred.

Striking was furthermore the clear predominance of female Erasmus incoming students. Almost 75% of all Erasmus-Week participants were female. The trend seen in many countries toward feminization of medicine was clearly evident among Erasmus incoming students. It can be speculated if female students are more willing to go abroad and take into account that this may lead to a prolonged duration of their studies, and if male students see a time-out at their home universities as an obstacle for their future career.

#### Pre- and post-questionnaire findings

Freetext comments given in the pre-questionnaires showed highly positive anticipation and curiosity of Erasmus incoming students concerning their stay abroad. On the other hand, concerns were mentioned regarding personal linguistic and professional abilities. Although for participation in the Erasmus programme a language certificate is mandatory, many Erasmus incoming students nontheless felt insufficiently prepared in their language proficiency. At the University of Leipzig, interdisciplinary language courses are offered that are open for all Erasmus incoming medical students and that are frequently booked by these students. Nontheless, the language courses seem to be regarded as an insufficient preparation for beginning medical studies. Support in intensifying language ability prior to the learning experience abroad is propagated extensively [7]; if these measures actually lead to alleviation of existing doubts remains unclear. For attenuation of such doubts and reservations, introduction of a course week supporting orientation and integration of incoming Erasmus students seemed justified.

The free-text question in the post-questionnaire asking if the Erasmus-Week should be offered to Erasmus incoming students in future was answered positively without exception. The possibility to combine German medical language knowledge acquisition and practice basic examination techniques in small groups with peer-teachers was emphasized multiple times as being extremely helpful and motivating.

The casual atmosphere in a protected environment together with German medical students was used as a platform to obtain support in general and Erasmus incoming students frequently stayed in contact with LernKlinik peer student tutors even after the course week. In some cases, informal tandem partnerships and mentoring relationships evolved after the course week. Future evaluations are planned to investigate if formal tandem partnerships should be initiated during Erasmus-Weeks. So far, support offers of the medical school dean’s office as well as the university’s academic foreign students’ office are available for Erasmus incoming students; yet, a structured mentorship programme offered by Leipzig medical students for Erasmus incoming students is not implemented so far, and may be developed on the basis of the Erasmus-Week described here.

Course compilation for the Erasmus-Week had several aims: on the one hand, hands-on skills courses from disciplines frequently selected by incoming Erasmus students were chosen, and concommitantly, courses dealing with interdisciplinary and clinically relevant basic skills procedures were selected. Taking these inclusion criteria into account, the compilation decribed in Figure 2 (Fig. 2) evolved, forming the basis for the Erasmus-Week structure of seven basic skills courses, supplemented by three further courses selected from the compilation of Figure 3 (Fig. 3) to amount to a total of ten courses. The number of courses was decided upon taking into account organizational and scheduling restrictions (avoiding time collisions with parallel language courses at the university campus, leaving sufficient free time to clarify questions on issues concerning life and study in Leipzig).

Grading of the individual courses showed that all courses offered every year (see Figure 2 (Fig. 2)) were marked with “very good” or “good” in over 70% of cases. Courses not being offered every year in the Erasmus-Week (see Figure 3 (Fig. 3)) showed “very good” or “good” markings in 70% as well, with the sole exception of the course in venipuncture. This raises the question as to how effectively evaluation results can contribute to course modification and optimization; a questionnaire distributed at the end of the Erasmus year to Erasmus incoming students seems necessary to obtain further insights as to how an orientation week should be ameliorated in future.

#### Further developments

The Erasmus-Week at the LernKlinik Leipzig is meanwhile a well-established component of elective course offers at this institution; the project has been described in the faculty reports published yearly [8] and presented at several conferences [9], [10] where it was wellreceived. For further qualitative improvement of the project, questionnaires distributed to peer student tutors giving the Erasmus courses are planned for collection of further ideas and proposals concerning the course week. Questionnaires distributed to Erasmus incoming students at the end of the entire Erasmus year are being planned to classify the relevance of the Erasmus-Week for the respective students. Collecting evaluations from Erasmus incoming students who decided not to participate in the Erasmus-Week may also be of interest. A survey focusing on the first cohort of 2012 is underway to elucidate possible connections between the stay abroad and future careers of former Erasmus incoming students. Results of this survey may serve as material for further development of the Erasmus-Week as well.

## Conclusions

Implementation of a preparatory course week for incoming Erasmus students focusing on language and practical clinical skills training using a peer-teaching method may alleviate commencement of studies at a medical faculty. If these acquired experiences influence future career orientation or workplace selection is to be elucidated in further studies. To conclude, we hope that this project underpins the following quote [2]:

“Erasmus has brought forth the first generation of young Europeans.”

(Umberto Eco, Italian writer)

## Acknowledgements

Special thanks to all peer-student-tutors of the LernKlinik who were highly engaged and motivated in the process of developing and enabling the Erasmus-Week, and handing over their knowledge and experiences to future peer student tutor generations.

## Competing interests

The authors declare that they have no competing interests. 

## Figures and Tables

**Table 1:**
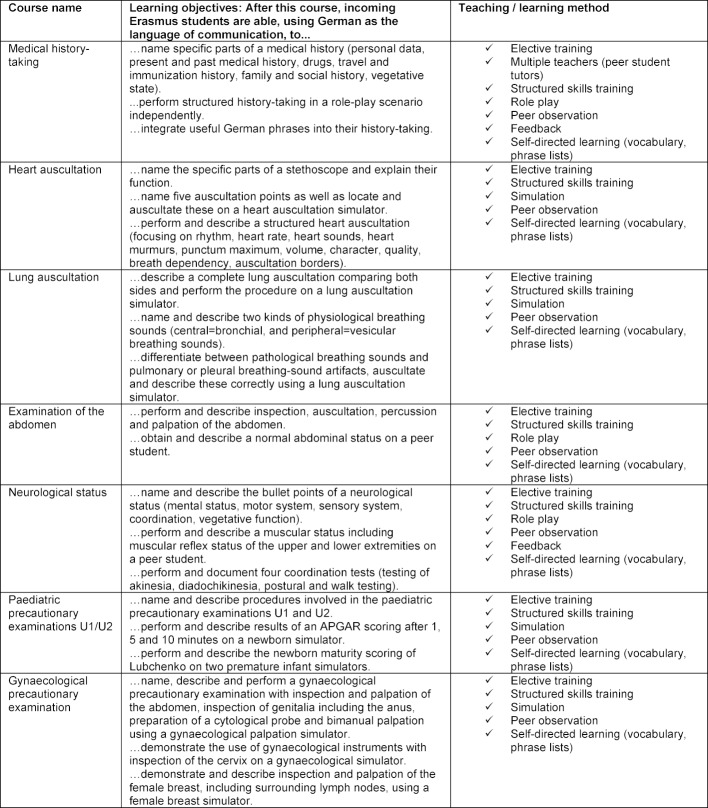
Compilation of the seven 90-minute Erasmus-Week courses given every year from 2012-2017, including learning objectives and teaching/learning methods

**Table 2:**
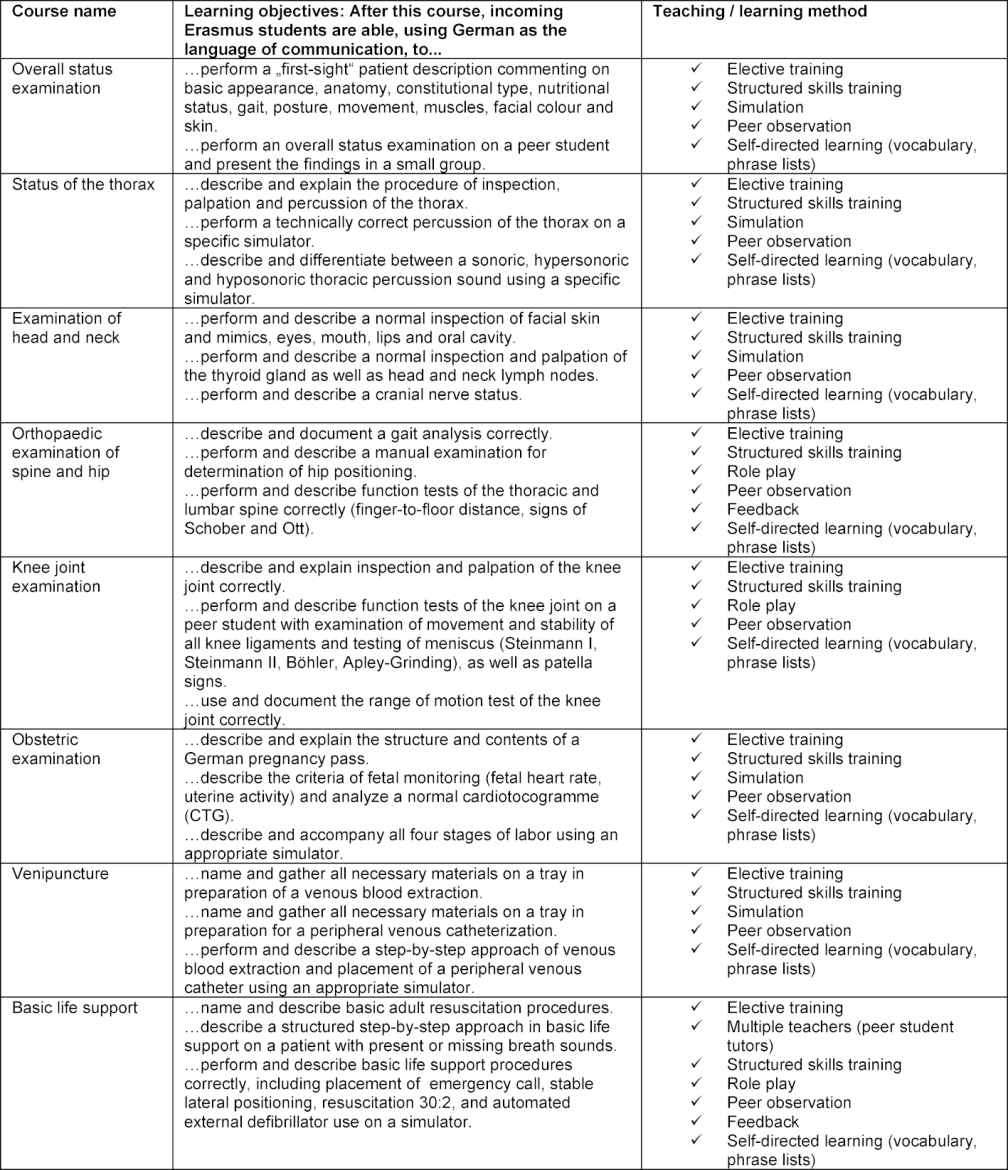
Compilation of 90-minute Erasmus-Week courses given intermittently between 2012 and 2017, including learning objectives and teaching / learning methods

**Table 3:**
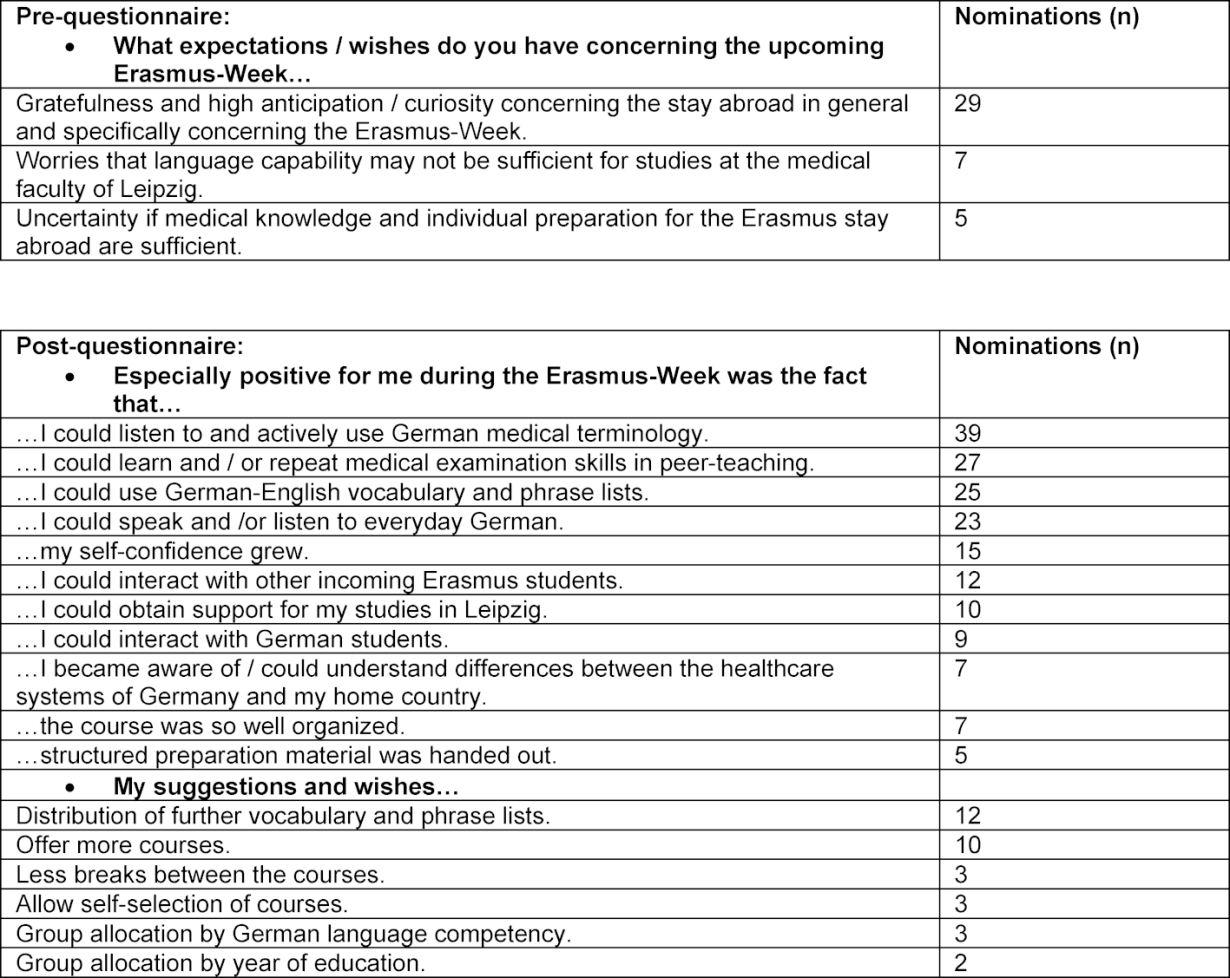
Distribution of qualitative pre- and post-questionnaire results among incoming Erasmus students between 2012 and 2017 (6 cohorts, n=148 participants)
